# Genomic Pathogen Typing Using Solid-State Nanopores

**DOI:** 10.1371/journal.pone.0142944

**Published:** 2015-11-12

**Authors:** Allison H. Squires, Evrim Atas, Amit Meller

**Affiliations:** 1 Department of Biomedical Engineering, Boston University, Boston, Massachusetts, 02215, United States of America; 2 Department of Biomedical Engineering, The Technion–Israel Institute of Technology, Haifa, 32000, Israel; University of Hyderabad, INDIA

## Abstract

In clinical settings, rapid and accurate characterization of pathogens is essential for effective treatment of patients; however, subtle genetic changes in pathogens which elude traditional phenotypic typing may confer dangerous pathogenic properties such as toxicity, antibiotic resistance, or virulence. Existing options for molecular typing techniques characterize the critical genomic changes that distinguish harmful and benign strains, yet the well-established approaches, in particular those that rely on electrophoretic separation of nucleic acid fragments on a gel, have room for only incremental future improvements in speed, cost, and complexity. Solid-state nanopores are an emerging class of single-molecule sensors that can electrophoretically characterize charged biopolymers, and which offer significant advantages in terms of sample and reagent requirements, readout speed, parallelization, and automation. We present here the first application of nanopores for single-molecule molecular typing using length based “fingerprints” of critical sites in bacterial genomes. This technique is highly adaptable for detection of different types of genetic variation; as we illustrate using prototypical examples including *Mycobacterium tuberculosis* and methicillin-resistant *Streptococcus aureus*, the solid-state nanopore diagnostic platform may be used to detect large insertions or deletions, small insertions or deletions, and even single-nucleotide variations in bacterial DNA. We further show that Bayesian classification of test samples can provide highly confident pathogen typing results based on only a few tens of independent single-molecule events, making this method extremely sensitive and statistically robust.

## Introduction

Subtle genetic changes in bacteria can produce large variations in factors affecting pathogenicity, such as toxicity, antibiotic resistance, and virulence. These genetic variations are not only used to trace the epidemic and phylogenetic relationships among strains of bacteria, but are also critically important in clinical settings for proper patient diagnosis and treatment. Most existing approaches require sample incubation and growth over the course of multiple days prior to testing, and nearly all require expert handling of samples and interpretation of results. Traditional phenotypic typing techniques such as serotypes, biotypes, phage-types, and antibiograms lack the necessary sensitivity to distinguish between closely related pathogen strains, and therefore fail to adequately capture these critical variations for clinical applications. Gel-based techniques such as restriction fragment length polymorphism (RFLP) or cleaved amplified polymorphic sequences (CAPS) require a large amount of time and results are not easily compared or transferred among labs. Next-generation sequencing is an increasingly popular method of fully characterizing bacterial strains [1] and may be used for typing strains according to the sequences of a panel of housekeeping genes, as in multi-locus sequence typing (MLST) [2], but this approach is more commonly used to trace *post hoc* epidemic and phylogenetic relationships among clinical isolates. Furthermore, the complexity and quantity of sequencing data far exceeds the minimum information required to efficiently and accurately diagnose a patient. For example, bioinformatics studies suggest that a panel of just 30–50 single nucleotide variations (SNVs) could be used to uniquely identify thousands of strains of Mycobacterium tuberculosis [3, 4]. Yet SNVs are not the only source of variation among pathogens; polymorphisms from SNVs and short indels up to genetic changes as large as whole plasmids or sets of genes may be responsible for critical changes to pathogenicity. Thus there exists a clear clinical need for a novel approach to molecular typing that can quickly and simply screen patient samples for a panel of widely varying known genetic polymorphisms of dangerous pathogens.

Solid-state nanopores may be used to discriminate the lengths of unlabeled individual biopolymers such as DNA molecules across a wide range of lengths [5, 6]. Biopolymers are electrophoretically attracted and threaded through a voltage-biased nanoscale pore drilled in an ultrathin freestanding SiN_x_ membrane [7, 8]. When a DNA molecule is threaded through a nanopore, it partially blocks the flow of ions moving through the pore, allowing real-time detection of the analyte by monitoring changes in the ion current. Nanopore sensing is biochemically simple, as it does not require labeling of the analyte with radioactive or fluorescent probes, yet it can be used to detect minute quantities of nucleic acid molecules, surpassing the sensitivity of bulk methods [8]. Moreover, nanopore sensing involves relatively simple instrumentation (primarily a current amplifier) and may be used to analyze thousands of molecules in just a few minutes, making this technique an ideal candidate for applications such as nucleic acid based diagnostics.

**Fig 1:**
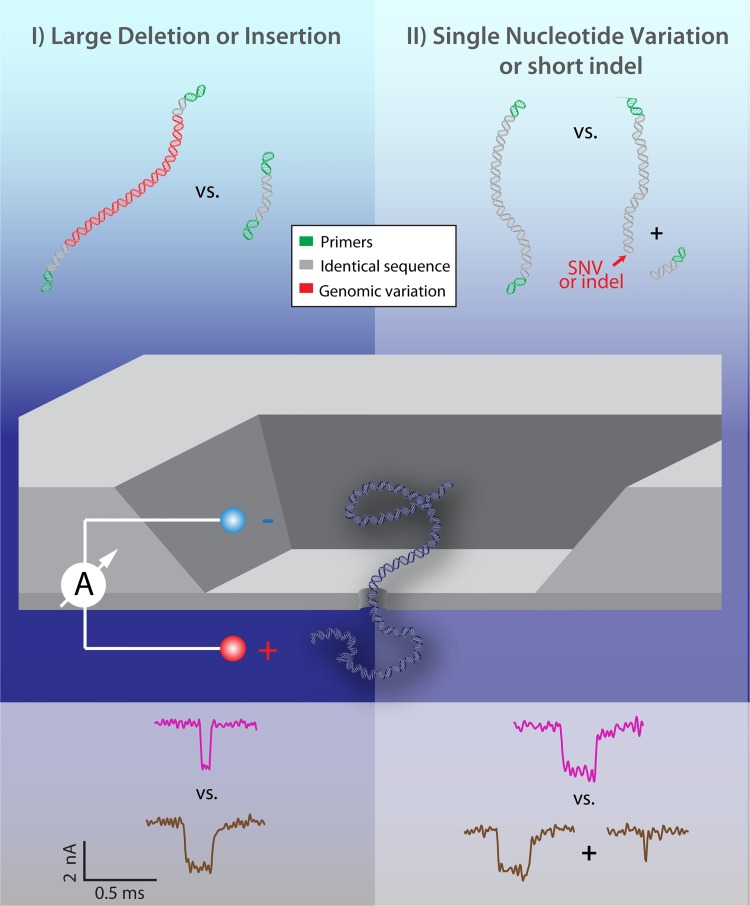
Two Principal Modes for Nanopore Discrimination of Pathogen Genomic Variation. Mode I: Direct length detection according to analyte translocation dwell time and depth enables discrimination of longer vs. shorter fragments; i.e: whether or not an insertion or deletion is present (left). Mode II: Prior to translocation, samples are exposed to a restriction enzyme that cuts at the site of a SNV or short indel or mutation. Detection of cleaved vs. uncleaved DNA fragments in the nanopore reveals whether or not the critical genomic variation is present.

Here we describe and practice a novel detection scheme (Fig 1) for molecular typing of pathogens using solid-state nanopores, and demonstrate its ability to discriminate a wide range of critical genetic polymorphisms in closely related organisms with starkly different pathogenicities. In the first sensing mode of our approach (Mode I), large insertions or deletions are detected by directly classifying the length of DNA in the nanopore. In the second sensing mode (Mode II), small indels down to SNVs may be detected by sequence-specific digestion at the site of the polymorphism to produce either one or two DNA fragments, which are then detected in the nanopore. We first characterize the practical range of our nanopore system for detecting variation in DNA length, and show that fragment length differences are more readily apparent for shorter DNA lengths and for asymmetric cut sites. We then demonstrate that statistical analysis tools such as Bayesian classifiers, commonly used for automated classification, are highly effective for rapid and statistically robust discrimination among different lengths and combinations of DNA fragments translocating through a nanopore, even in cases where significant portions of these distributions overlap. We apply these techniques to demonstrate polymorphism discrimination down to the single nucleotide level in prototypical strains of *Mycobacterium tuberculosis* (virulent vs. avirulent) and *Streptococcus aureus* (methicillin-resistant vs. multi-drug resistant). This highly versatile combination of rapid length and digest discrimination, spanning several orders of magnitude of possible genomic variation size, in a single, parallelizable device, could be extended to probe a large panel of critical sites within a genome for point-of-care determination of critical pathogenic properties and sequence typing.

## Results and Discussion

### Detection of DNA Sequence Polymorphisms in Solid-State Nanopores

The simplest form of nanopore translocation analysis involves the measurement of the depth of each current blockade (Δ*I*_*B*_) and the dwell time of each molecule within the pore (*t*_*D*_). Both parameters have been shown to grow nonlinearly with DNA length, forming the basis for fragment length separation in the nanopore system. The statistical distributions of these independently measured quantities may be used to distinguish between analytes of different lengths, such as DNAs [5, 6, 9], or proteins having identical molecular weight but slightly different charge or 3D structure [10–13]. Variation in the translocation dwell-time (*t*_*D*_) in solid-state nanopores measured for different DNA lengths (*l*), are empirically described by a power law: *t*_*D*_ ∼ *l* ^*α*^ where *α* = 1.38±0.02, which has been reproduced by multiple experimental approaches [5, 9, 14]. Using a log-scale distribution of translocation times to estimate the distribution of *t*_D_, note that the difference in log(*t*_D_) for two sequences (lengths *l*_0_ and *l*_0_ + Δ*l*) is more apparent for shorter length *l*_0_ as compared with the insertions and deletions Δ*l* (*i*.*e*. when Δ*l*/*l*_0_ ∼ 1) according to Eq 1:

$$\Delta \;\text{log}(t_{D})∼\alpha \;\text{log}(1+\frac{\Delta l}{l_{0}})$$ (1)

If the presence of two fragment lengths must be identified from within a single sample, it is desirable that their distributions of Δ*I*_*B*_ or *t*_*D*_ should be as well-separated as possible. Furthermore, if the presence of a cut sample must be distinguished from an uncut sample, then by Eq 1 the peak produced by the shorter part of a cut sample will appear farther away from the uncut peak than the longer part of a cut sample. To statistically distinguish the samples, it is desirable for the peak of the shorter part to be as dissimilar as possible from the uncut peak. Therefore, asymmetrically cut DNA pieces from a restriction digest are more readily distinguished from the original uncut length than those produced by symmetrically positioned restriction sites, provided that the shorter piece is of sufficient length to be detected by the nanopore. In cases where separation between two similar length biopolymers (Δ*l*/*l*_0_ ∼ 1) is required, the measured histograms of either Δ*I*_*B*_ or *t*_*D*_ may overlap significantly, making discrimination between these molecules difficult. Combinations of multiple fragment lengths within a sample pose additional challenges, as their more complicated distributions may overlap or otherwise preclude simple contour cluster separation.

In the context of sequence typing, identification of fragments by sizing will indicate the presence of specific insertions and deletions that may enhance or reduce pathogenicity or otherwise uniquely identify a pathogenic strain. Upper bounds on Δ*l* are set by: 1) sample preparation parameters and limitations; for example, robust and fast PCR amplification is most easily achieved for fragment lengths of ~10^2^–10^3^ bp [15] and 2) nanopore stability considerations; for example, nanopores are more frequently clogged by very long DNA (>20 kbp). Lower bounds on *l*_0_ are set by nanopore sensitivity; while several groups have demonstrated detection of small DNA fragments (<50 bp) [16] we find that a minimum *l*_0_ on the order of ~100 bp is more reliable since it is readily detectable in small nanopores with no additional modifications [5], producing an extremely small fraction of missed events due to the finite system bandwidth. Thus a reasonable design range for sequence typing fragments is ~100 bp minimum length for *l*_0_, ranging up to a few thousand base pairs maximum length for *l*_0_ + Δ*l*. Many types of common genetic variations used for strain typing fall within this size range. For example, one complete IS6110 (insertion-like sequence element) insertion in *M*. *tuberculosis* is 1358 bp [17]. At the other end of this range, multi-drug resistant strains of methicillin-resistant *S*. *aureus* (MRSA) have many insertions and deletions in the range 47 bp—643 bp that affect their pathogenicity [18]. To detect the smallest indels, which fall below the minimum detectable Δ*l*, we turn to the exquisite sequence specificity of digestion by restriction enzymes, which can identify sequence polymorphisms down to a single nucleotide variation.

Using these design principles, we present here two alternative modes of detection that illustrate the wide range of genomic variations that may be detected using a single sensor. For large insertions or deletions (Fig 1: Mode I, left panel), a nanopore may be used to discriminate the raw change in DNA length caused by the presence or absence of this sequence according to the duration of translocation events. For short indels, mutations, or single nucleotide variations (SNVs) (Fig 1: Mode II, right panel), which are more difficult to identify solely by length as discussed above, we utilize a restriction enzyme. The sample is only cut in the presence (or absence) of the critical sequence, and subsequent detection in a nanopore reveals either one or two fragments in the nanopore according to the observed durations and blockage levels of translocation events.

### Event Diagram Discrimination of Sample Length and Composition

**Fig 2:**
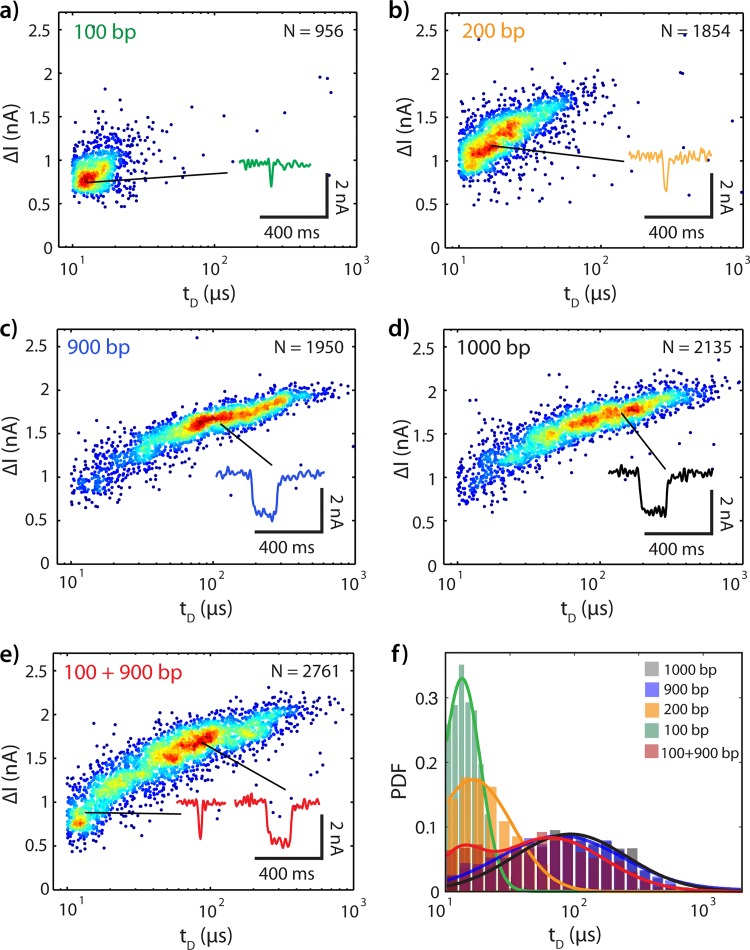
Translocation Event Diagrams Uniquely Identify DNA Fragment Lengths in a Single Nanopore. (a) 100 bp at 1 nM. (b) 200 bp at 1 nM. (c) 900 bp at 1 nM. (d) 1000 bp at 1 nM. (e) 1:1 combination of 100 bp and 900 bp, total concentration 2 nM. (f) Semilog(x) distributions of translocation dwell times for all samples (a)-(e). Translocations for all samples were collected in a single nanopore (4.8 nm diameter, effective thickness ~7 nm) with a +300 mV bias relative to *trans* (open pore current: 13 nA). To facilitate visualization of population density, a random white noise offset below the acquisition rate of this data (-2 μs < Δ*t* < +2 μs, acquisition rate 250 kHz) has been added to each *t*_D_.

We first experimentally illustrate the practical length resolution of the nanopore platform for identifying sample length and composition. We analyzed samples containing mixtures of DNA fragments composed of one or two well-defined lengths. The resulting event diagrams create unique fingerprints that can be used to distinguish different lengths of DNA (Mode I) or whether or not a fragment of DNA has been cut (Mode II). Fig 2A–2E show event diagrams for 100 bp, 200 bp, 900 bp, 1000 bp, and 100+900 bp DNA in a single nanopore (diameter 4.8 nm, effective height 7 nm) at +300 mV bias (for additional examples, see Figs B-E in S1 File). Here, each translocation event is represented by its corresponding ion current event amplitude (Δ*I*_*B*_) and dwell time (*t*_*D*_). From comparison of Fig 2A and 2D, it is evident that insertions and deletions Δ*l* several times larger than the base length (here: Δ*l*:*l*_0_ = 9:1) are indeed easily distinguishable (Fig C in S1 File). Comparison of Fig 2A and 2B illustrates that Δ*l* = 100 bp results in reasonably distinct event diagrams for *l*_0_ = 100 bp, which may be distinguished to >95% confidence with just a few events each, taking both dwell time and current amplitude into consideration (Fig D in S1 File). However, at *l*_0_ = 900 bp a minimum of several hundred events are required to confidently (>95%) differentiate *l*_0_ (Fig 2C) from *l*_0_ + Δ*l* (1000 bp, Fig 2D), since their event diagrams overlap significantly (Fig E in S1 File). Returning to Eq 1, for Δ*l* = 100 bp, we expect Δlog(*t*_*D*_) = 0.415 for *l*_0_ = 100 bp, and Δlog(*t*_*D*_) = 0.063 for *l*_0_ = 900 bp. For the data shown in Fig 2F, Δlog(*t*_*D*_) = 0.1 for *l*_0_ = 100 bp, and Δlog(*t*_*D*_) = 0.03 for *l*_0_ = 900 bp. The inability to easily and quickly discriminate the 900 bp DNA from the 1000 bp DNA demonstrates the practical limits set on Mode I sample identification according to the size of the insertion or deletion that must be detected.

Fig 2E illustrates how Mode II may overcome these limitations by digesting DNA into fragments: here, a highly asymmetric ratio of lengths in a mixed sample (100+900 bp) clearly facilitates sample identification as compared to the full length 1000 bp DNA (Fig 2D). However, Mode II also presents a more challenging case for quantitative discrimination between an uncut and a cut sample. Whereas single-length samples can be identified using either their *t*_*D*_ or *I*_*B*_ distribution (as shown in Fig 2F), the longer fragment in a cut sample may share significant overlap with the uncut sample. This is particularly true in the case of a highly asymmetric cut site.

### Bayesian Statistical Treatment of Nanopore Data

We seek to develop a statistically robust and data processing procedure for nanopore-based discrimination among DNA fragment lengths. We note that in diagnostic tests, biochemical assays are ideally designed to classify samples among just a few known possible cases, rather than to produce a continuously-valued scalar associated with a physical quantity of the system (*i*.*e*. mean translocation dwell-time, or event amplitude). To quantitatively identify unknown samples, which must fall into one of two (or a few) known possible genetic variation classifications at each relevant locus, we have developed a classification framework for nanopore sensing based upon Bayesian classification [19]. This approach enables surprisingly rapid identification of unknown samples, and more importantly, provides an estimate of the statistical confidence associated with these classifications.

The event diagrams shown in Fig 2 may be modeled as probability density functions *Z*_i_ describing the likelihood of observing a translocation with depth *I*_*B*_ and dwell time *t*_*D*_. Here, we find the maximum likelihood fit of a Gaussian Mixture Model (GMM) to the event diagram to describe each distribution as a sum of one or more two-dimensional covariant Gaussian probability density functions (Eq 2).

$$\begin{array}{l} p\left(\theta_{\text{model}}|Z\right)=\sum_{k}w_{k}\cdot g(\theta_{\text{model}}|\mu_{k},\Sigma_{k})\\ \text{where}:\;g(\theta_{\text{model}}|\mu_{k},\Sigma_{k})=\frac{1}{2\pi \left({\left|\Sigma_{k}\right|}^{1/2}\right)}\text{exp}\left[\frac{-\left(\theta_{\text{model}}-\mu_{k}\right)'\Sigma_{k}{}^{-1}\left(\theta_{\text{model}}-\mu_{k}\right)}{2}\right] \end{array}$$ (2)

Here, *p*(**θ**_model_|*Z*) is the GMM for case *Z* for the model data set **θ**_model_ (here: variables *I*_*B*_ and *t*_*D*_) summed over all *k* components, *w*_*k*_ is the relative weighting of each component such that $\sum_{k}w_{k}=1$, *μ*_*k*_ is the mean of each component, and ***Σ***_*k*_ is the covariance matrix of each component. We then calculate the posterior likelihood, *p*(*Z*_*i*_|**θ**), that an unknown sample translocation data set, **θ**, belongs to the *a priori* distribution *Z*_*i*_ according to Bayes’ rule (Eq 3):

$$p\left(Z_{i}|\theta \right)=\frac{p\left(\theta |Z_{i}\right)\cdot P(Z_{i})}{\sum_{j}p\left(\theta |Z_{j}\right)\cdot P(Z_{j})}$$ (3)

The “unknown” sample is then assigned to whichever *Z*_*i*_ yields the higher posterior likelihood. The posterior likelihood also represents the expected accuracy of the resulting decision. Here, the prior probabilities are taken to be equal, *P*(*Z*_*i*_) = 0.5. In a clinical application, the prior probabilities may be adjusted to account for the known incidence of each possible case in the clinical population under consideration, and additional weighting factors may be included to bias the classifier and thereby avoid costly diagnostic errors.

**Fig 3:**
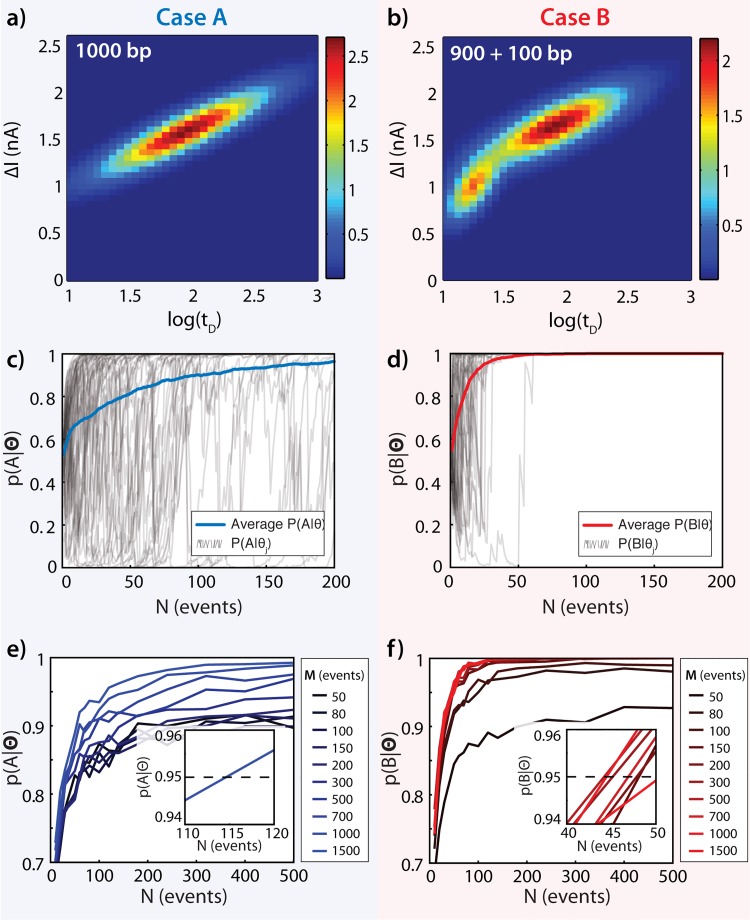
Gaussian Mixture Models for Mode II Classification of 1000 bp vs. 900+100 bp DNA Fragments. (a) 2-D GMM for 1000 bp DNA fragment translocations. (b) 2-D GMM for 900+100 bp DNA fragment translocations. (c) Bayesian posterior estimates *p*(*A*|**Θ**) of correctly identifying a data set **Θ** as Case A, calculated for each increment of *N* points in **Θ**, repeated 1000 times (first 50 shown in gray) and averaged (blue), each using *M* = 1500 points in the model data set. (d) Bayesian posterior estimates *p*(*B*|**Θ**) of correctly identifying a data set **Θ** as Case *B*, calculated for each increment of *N* points in **Θ**, repeated 1000 times (first 50 shown in gray) and averaged (red), all using *M* = 1500 points in the model data set. (e) Bayesian posterior estimates *p*(*A*|**Θ**) for test data sets of *N* points given a model based on data set size *M*. Each point represents the average of 1000 separate bootstrap simulations. (f) Bayesian posterior estimates *p*(*A*|**Θ**) for test data sets of *N* points given a model based on data set size *M*. Each point represents the average of 1000 separate bootstrap simulations. Insets: range of *N* for which *p*(*A*|**Θ**) reaches 0.95. See Methods and S1 File for complete numerical simulation details.

Fig 3 illustrates the utility of this approach for discrimination between probability density functions with significant overlap, as would be the case for sensing Mode II (Fig 1). Fig 3A and 3B show the maximum-likelihood Gaussian Mixture Model fits to the event diagrams for enzymatically “uncut” DNA and for “cut” DNA. The color temperature indicates the relative probability density of the model at each location. Fig 3A shows the GMM for 1000 bp DNA (Case A), which represents a whole fragment that does not contain the critical sequence for digestion by a particular restriction enzyme. This produces a single population on the Gaussian mixture model fit. In Fig 3B, a 100+900 bp DNA equimolar mixture represents a restriction-digested sample that was originally 1000 bp long, with the critical digestion site located at 100 bp from one end of the DNA (Case B). This mixture shows two clear populations in the GMM, one matching the 100 bp event diagram fingerprint (Fig 2A), and one matching the 900 bp event diagram fingerprint (Fig 2C), which has significant overlap with the 1000 bp GMM in Fig 3A. Notably, the GMM fit for this two-component sample is readily distinguishable from the full-length 1000 bp sample due to the clear peak at short timescales caused by the 100 bp fragment.

The confidence with which an unknown sample may be classified increases with the number of translocations collected, *N*. Fig 3C and 3D depict the posterior probabilities *p*(*A*|***Θ***) or *p*(*B*|***Θ***) for *correctly* assigning an unknown sample to either case *A* (uncut, 1000 bp) or case *B* (cut, 100+900 bp) for *N* translocations of the unknown sample. Each light gray curve represents a single simulation run of the posterior probabilities calculated after *N* translocations of the unknown sample, using a randomly selected training set of 1500 translocations for the GMM fit, and a randomly selected and ordered test data set from the remaining translocations (for clarity, only the first 50 simulation runs are shown here). The red and blue curves represent the average posterior probabilities for correctly identifying an A sample and a B sample, respectively, averaged over all 1000 simulation runs. These clearly highlight a general trend emerging from our data: a rapid increase in the probability for *N* of just a few tens of events from the unknown test sample, followed by a slow increase for larger values of *N*.

**Fig 4:**
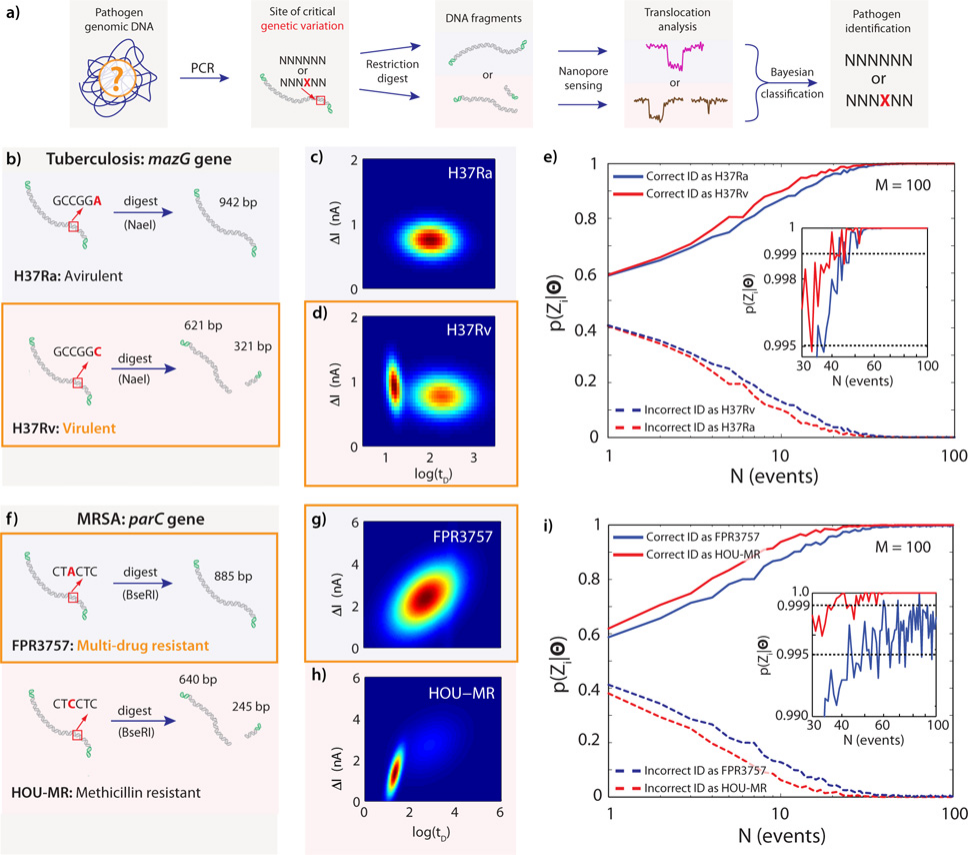
Gaussian Mixture Models of DNA Fragments for Actual Mode II Pathogen Typing at the SNV Level. (a) Diagram of the main steps in sample preparation, detection, and classification: PCR fragments from isolated pathogens are subjected to a restriction digest, which recognizes and cuts only one genomic variant. Nanopore translocations are used to classify the pathogen according to the combination of fragment lengths detected. (b) The *mazG* gene of the avirulent *M*. *tuberculosis* strain H37Ra is not cut by NaeI (942 bp), while the same gene in the closely related virulent strain H37Rv, which differs by only a single A-to-C mutation, is cut by NaeI (621bp + 321 bp). (c) Gaussian mixture model (one component) fit to translocations of *mazG* fragments from H37Ra. (d) Gaussian mixture model (two components) fit to translocations of *mazG* fragments from H37Rv. (e) Posterior probabilities for correctly identifying the H37Ra and H37Rv strains as a function of number of translocation events collected from an unknown sample, simulated using bootstrap sampling from nanopore translocation data. (f) The *parC* gene of the multi-drug-resistant MRSA strain FPR3757 is not cut by BseRI (886 bp) due to a single C-to-A mutation, while the closely related and less resistant strain HOU-MR is cut by BseRI (640bp + 245 bp). (g) Gaussian mixture model (one component) fit to translocations of *parC* fragments from FPR3757. (h) Gaussian mixture model (two components) fit to translocations of *parC* fragments from HOU-MR. (i) Posterior probabilities for correctly identifying the FPR3757 and HOU-MR strains as a function of number of translocation events collected from an unknown sample, simulated using bootstrap sampling from nanopore translocation data.

The rate of convergence and maximum confidence of the posterior probability curves shown in Fig 3C and 3D are also dependent upon the number *M* of translocations used for fitting the GMM. Fig 3E and 3F show posterior probability curves for *A* and *B* for a range of model data set sizes, *M*. It is clear that larger training sets are necessary to achieve high levels of confidence; in this case, if fewer than M = 700 samples make up the training sets, the confidence level of correctly identifying the cut fragments does not rise above 95%, even for relatively large *N* = 500 translocations. However, the number of points *M* required in the model set varies widely depending upon the similarity of the distributions to be discriminated: for example, for the distributions shown in Fig 4, only about *M* = 100 data points are necessary to exceed 95% confidence with only *N* = 50 test points (Fig G in S1 File). Insets show the number of translocation events required for each posterior probability to reach a 95% confidence threshold (N = 45 events and N = 115 events for identifying an A sample or a B sample, respectively). These curves allow quantitative determination of how many translocations from an unknown sample should be collected in order to verify the sample identity with any desired level of confidence for any pair or set of possible cases under consideration. Surprisingly, we find here that *only a few tens of events are necessary to confidently classify an unknown cut or uncut sample*. At typical nanopore collection rates (~1 event/second for ~1 nM DNA) less than a minute would be sufficient to obtain a confident result. Additionally, the curves shown in Fig 3C–3F illustrate that the limiting case (for this example) is actually the uncut sample; that is, more translocations from an unknown sample are required to confidently identify a sample as *A* (1000 bp) rather than *B* (900+100 bp).

### Pathogen Discrimination Sensitivity Down to Single-Nucleotide Variations

We demonstrate here the highest sensitivity of our proposed analysis by discrimination of critical single nucleotide variations in actual pathogen genomes, selected at sites that are known to alter pathogenicity. We selected two model pathogens with very different characteristic phenotypic effects caused by a lone SNV: *S*. *aureus*, in which a SNV confers antibiotic resistance via the *parC* gene, and *M*. *tuberculosis*, in which a SNV affects virulence via the *mazG* gene. The emergence of methicillin-resistant *S*. *aureus* (MRSA) strains in recent years has increased the incidence of severe nosocomial and community-acquired infections. The wide variation in antibiotic resistance across this set of pathogens necessitates either immediate treatment with broad-spectrum antibiotics that can perpetuate the evolution of resistant strains, or accurate but expensive and time-consuming pathogen characterization prior to treatment, or (often) both. MRSA exhibits a relatively high degree of genetic variation due in large part to frequent horizontal gene transfer [20]. Antibiotic resistance in strains of MRSA is often conferred by mobile genetic elements (MGEs), which are large insertions or deletions that can contain one or more whole genes from related bacteria or phages [21]. MRSA also exhibits frequent small mutations and indels [18]. In contrast, *M*. *tuberculosis*, the bacterium responsible for tuberculosis (TB), exhibits relatively little genetic diversity among its various strains [22] but has rapidly increasing incidence of antibiotic-resistant and hypervirulent strains.

For each of these two prototypical pathogens we have selected two well-studied and closely related strains to illustrate the sensitivity of our technique by sensing single critical SNVs that differentiate the pathogens. For methicillin-resistant *S*. *aureus*, we selected two closely related isolates of the well-characterized USA300 strain, USA300-FPR3757 and USA300-HOU-MR. Both of these isolates are methicillin-resistant, but otherwise possess very different spectra of antibiotic resistance [18, 23] due to variations in plasmids, MGEs, and many other smaller indels and SNVs. For *M*. *tuberculosis*, we selected closely related virulent (H37Rv) and avirulent (H37Ra) strains, which differ only in several key SNVs and indels [18]. This pair has been widely used as reference strains for studying virulence and pathogenesis of *M*. *tuberculosis*, and H37Ra is even used to boost immunogenicity during TB immunization.

Fig 4A shows the main steps of sample preparation required to amplify and digest each locus of interest from a pathogen sample. For each pathogen, we focused on specific genes where single nucleotide variations are significantly correlated with the pathogenicity of the strains: The *mazG* gene in *M*. *tuberculosis* encodes an NTP pyrophosphohydrolase, which promotes cell viability under oxidative stress [24]. A single nucleotide variation of C to A in *mazG* at location 239 bp in H37Ra is believed to be critical to the original emergence of this less virulent strain (as compared to the original H37Rv from which it derives) by conferring a competitive advantage during aging-mediated cell lysis [25]. We designed primers and obtained an amplicon of 942 bp for this gene. As shown in Fig 4B, digestion was performed with NaeI (NEB) to produce fragments of 321 bp and 621 bp for H37Rv. The amplicon from H37Ra was subjected to the same digestion protocol, but did not contain a cut site and therefore remained full-length (942 bp). We confirmed the amplification of the *mazG* gene and its subsequent digestion by running native PAGE gels, as shown in S1 File. Fig 4C–4D show the GMM fit for the amplified fragment from H37Ra and its counterpart from H37Rv. The GMM fit for the cut H37Rv sample is clearly distinguishable from that of the uncut H37Ra. Based on the bootstrap simulations shown in Fig 4E and 4F for Bayesian classification posterior estimates as a function of test data set size *M*, these *M*. *tuberculosis* strains can be distinguished with >99.5% confidence in *N* = 50 translocations or fewer, using a model data set size of only ~100 points (see Fig G in S1 File for additional numerical simulation data).

For the MRSA strains, we chose the *parC* gene (2.4 Kb) and amplified a subregion that includes a critical SNV site. While both strains are methicillin resistant, the FPR3757 shows a much larger spectrum of antibiotic resistance [26]. Specifically, the SNV in the FPR3757 parC gene (C to A) is believed to confer fluoroquinolone resistance. Amplicons (885 bp) from these strains were digested with BseRI, as shown in Fig 4F. The amplicon from the HOU-MR strain will produce two fragments upon digestion: 245 bp and 640 bp. The amplicon from FPR3757 does not have the cut site for BseRI, so it remains uncut (885 bp) (see Fig A in S1 File). Fig 4G and 4H show the GMM fits for the digested samples from FPR3757 and HOU-MR, respectively. Based on the bootstrap simulation shown in Fig 4I for Bayesian classification posterior estimates as a function of test data set size, this SNV can be used to discriminate between FRP3757 and HOU-MR with >99.5% confidence in N = 80 translocations or fewer, based on a model data set size of only ~100 points (see Fig H in S1 File for additional numerical simulation data).

A critical aspect of the approach outlined here is careful sample design to allow efficient sample identification at each site under consideration. Both Mode I and Mode II sensing depend upon differences in translocation time and blockage level caused by differences in sample length. In addition to PCR design of sample length, these variables are controlled by a multitude of factors, including pore geometry, material, and functionalization, electrical bias, and buffer conditions. Mode II sensing is additionally dependent upon the location of the restriction site within a fragment; asymmetric sites may produce a more obvious change in translocation dwell time or in blockage level, but very short fragments may produce dwell times below the sensing resolution of the nanopore, leading to “missed” events. These factors can be balanced for any given nanopore system to enable both Mode I and Mode II sensing and minimize the possibility for missed events. Note that for Mode I type sensing, the restriction digest is eliminated, conserving both time and resources. However, Mode II type sensing is more suitable to discriminate critical single nucleotide variations, which alter pathogenicity, and also proves the high sensitivity of our method.

## Conclusion

Solid-state nanopore based biosensing is a rapidly growing field due to its practical and conceptual simplicity, portability and versatility. To date, few reports have demonstrated the utility of the method towards clinical diagnostic applications. Yet as we have shown here, nanopores are well-suited to make statistically robust diagnostic classifications among different DNA lengths with real single-molecule data, even in cases where the distributions significantly overlap. Utilizing a Bayesian statistical model, we have demonstrated that nanopore sensing can be used to discriminate among pathogens based on well-known genomic variations. Both large indels (Mode I) or short indels and single nucleotide variations (Mode II) can be targeted using proper sequence-specific digestion with off-the-shelf restriction enzymes. Furthermore, the Bayesian classifiers indicate the statistical confidence of each classification as a function of the number of nanopore events obtained in each measurement. Even at this preliminary stage of development we find that only a few tens of events (obtained in just a few minutes using a single pore) are sufficient to produce a statistically reliable result with well-defined and small error margins.

Our method is general and can be adapted to address many different “multiple-choice” clinical questions using a nanopore biosensor or other single molecule approaches. Future extensions of this work may seek to design and implement large panels of critical sites that represent the minimum sets necessary to characterize genomic variation for various applications in healthcare and research, and to develop additional sensing modalities. Although the primary design challenge currently remains linked to the location and availability of restriction digestion sites, we expect that the ongoing development of designer restriction enzymes, for example systems based on modular zinc fingers [27], TALENs [28], or CRISPR-like proteins will provide additional design flexibility for this technique.

The nanopore fingerprinting approach presented here addresses clear needs in clinical molecular diagnostics for a rapid and simple sensor that can identify a wide range of genomic variation in pathogens to inform treatment options. We have shown here discrimination of both large and small scale genomic variations between pathogen strains, down to single SNVs. The large, flexible sample design space for lengths, cut sites, and enzyme selection at each critical locus ensures that the technique is highly customizable for different genomic variation panels that could profile pathogenicity, antibiotic resistance, or even sequence type. The inherent scalability, minimal sample requirements, speed, and simple readout of the nanopore platform would all facilitate on-site and perhaps even automated use: As successive events are recorded, an increasingly clear fingerprint of translocation times and blockage levels will permit online software to “call” the sample as soon as enough events have been accumulated. Our technique is highly portable and customizable, and the binary data would be readily transferrable among different labs.

## Materials and Methods

### Chip and Nanopore Fabrication

Nanopore chips were fabricated on a 4” silicon wafer coated with SiO_2_ (500 nm) and low-stress amorphous silicon nitride (SiN_x_, 60 nm). The SiN_x_ was locally thinned to <10 nm (1.5–2 μm circular wells) using a controlled RIE etch. Freestanding membranes of SiN_x_ (60x60 μm) were created by through-etching the wafer with KOH, with the locally etched wells aligned to the etched freestanding SiN_x_ membranes. Nanopores were fabricated in the thinned SiN_x_ regions using a high resolution TEM (Jeol 2010F), as previously reported [29]. Pore formation proceeded with visual feedback by iterating through a uniformly expanded beam for imaging the nanopore diameter during formation and converging the beam to locally sputter and melt the membrane. Pores of 4 ± 0.2 nm could be consistently formed.

### Sample Preparation

100 bp, 900 bp and 1 kbp, dsDNA was purchased from Fisher Scientific (NoLimits™ DNA) and used without further purification. *M*. *tuberculosis* strains H37Ra (ATCC®25177) and H37Rv (ATCC®25618) were freshly obtained from American Type Culture Collection (ATCC). In order to obtain the *mazG* gene from both *M*. *tuberculosis* strains, we designed primers for PCR amplification (35 cycles, denaturation 98°C / 10s, annealing 65°C / 30s, extension 72°C / 30 s; final extension, 72°C / 5 min) using New England Biolabs Phusion polymerase. The same protocol was used for both strains using the same primers. We chose a restriction enzyme specific to the single nucleotide variation: for the *mazG* gene in H37Rv, NaeI (New England Biolabs) will cut the amplicon into two pieces of 321 and 621 bp, whereas the *mazG* gene from H37Ra will not be digested with this enzyme (942 bp). Two different strains of methicillin-resistant *S*. *aureus* (USA300-HOU-MR; ATCC®BAA-1718 and USA300-FPR3757; ATCC®BAA-1556) were used in order to distinguish a single nucleotide variation in the *parC* gene. The amplicon for the MRSA *parC* gene was digested with the restriction enzyme BseRI (New England Biolabs), which cut the HOU-MR fragment into two pieces (245 bp and 640 bp), while the FPR3757 fragment remained full-length (885 bp). PCR for the MRSA strains was performed under similar conditions as for the *M*. *tuberculosis* gene amplification. Sequences and further details for these specific genes and corresponding PCR primers are provided in S1 File.

### Data Acquisition and Analysis

Electrical NP measurements were performed in a dark, double-insulated Faraday cage using our custom cell (described elsewhere) [30]. The ion current was measured using an Axopatch 200B (Molecular Devices) at 100 KHz bandwidth, sampled at 250 kHz using a National Instrument card. Data was acquired and analyzed using custom LabView codes. Numerical simulations and data analysis were all performed using Matlab (MathWorks) and Igor Pro (Wavemetrics).

### Numerical Simulations

Posterior probabilities were estimated using bootstrap resampling without replacement on nanopore translocation data (>2000 events per data set). Reported posterior probabilities are the average of many (typically >1000) iterations of this method. For each iteration, randomly selected disjoint subsets were selected from the original data sets *dataA* and *dataB* to represent a model set (*modelA* and *modelB*, size *M*) a test set (***Θ***_A_ and ***Θ***_B_, size *N*). 2-D Gaussian mixture models (*A*_fit_ and *B*_fit_) with either one (uncut fragment) or two components (cut fragments) were fit to each model set by expectation maximization, yielding fit parameters *μ* (component means), ***Σ*** (component covariance matrices), and *w* (mixture weights for each component). Posterior probabilities p(*A*_fit_|***Θ***_A_) (correct ID of ***Θ***_A_ as type A data), p(*B*_fit_|***Θ***_B_) (correct ID of ***Θ***_B_ as type B data), p(*B*_fit_|***Θ***_A_) (incorrect ID of ***Θ***_A_ as type B data), and p(*A*_fit_|***Θ***_B_) (incorrect ID of ***Θ***_B_ as type A data were calculated using these fits according to Eq 3, then averaged across all iterations. All simulations assumed equal prior probabilities for A and B (0.5). Additional analysis and numerical simulations are included in S1 File.

## Supporting Information

S1 File Supporting Information and Figures. Gene and primer sequences for tuberculosis and MRSA strains. Additional details for PCR and restriction digest (Fig A). Bayesian classification for Mode I: Additional data and numerical simulations (Figs B-E). Bayesian classification for Mode II: Additional data and numerical simulations (Fig F). *M*. *tuberculosis* and methicillin-resistant *S*. *aureus* SNV detection: Additional data and numerical simulations (Figs G and H). **Fig A in S1 File. PCR and Restriction Digest Products.** Native PAGE showing successful PCR amplification of the *mazG* gene (*M*. *tuberculosis*) and *parC* gene (*S*. *aureus*). Digestion reactions yield either cut or uncut amplicons depending upon the parent strain. Lane 1: loading dye. Lane 2: 100 bp NEB ladder. Lane 3: *mazG* gene amplified from H37Ra (942 bp). Lane 4: *mazG* gene amplified from H37Rv (942 bp). Lane 5: *mazG* from H37Ra after digestion with NaeI enzyme, not cut. Lane 6: *mazG* from H37Rv after digestion with NaeI, cut into two fragments of 321 and 621 bp. Lane 7: *parC* gene fragment amplified from HOU-MR strain (885 bp). Lane 8: *parC* gene fragment amplified from FPR3757 strain (885 bp). Lane 9: *parC* from HOU-MR after digestion reaction with BseRI, cut into two fragments of 245 and 640 bp. Lane 10: *parC* from FPR3757 after digestion reaction with BseRI, not cut. Digestion reactions were performed at 37°C for 1hr in NEB Cutsmart buffer. 10 units of enzyme were used for each digestion reaction. **Fig B in S1 File. Gaussian Mixture Model Fits for DNA Translocation.** Gaussian mixture model fits to translocations of single-length DNA samples through a 4.8 nm diameter nanopore (1M KCl, +300 mV bias). (a) 100 bp NoLimits DNA. (b) 200 bp NoLimits DNA. (c) 900 bp NoLimits DNA. (d) 1000 bp NoLimits DNA. Raw *t*_D_ and Δ*I* data are shown in Fig 2 (main text). **Fig C in S1 File. Bayesian Posterior Estimates for Nanopore Sample Identification.** Bayesian posterior estimates *p*(100bp|Θ) and p(1000bp|Θ) for test data sets of *N* points given a model based on *M* points. Data is bootstrapped from translocations of (a) 100 bp NoLimits DNA and (b) 1000 bp NoLimits DNA (main text: Fig 2A and 2D) corresponding to the Gaussian Mixture Models shown in Figs Ba and Bd. Each point represents the average of 1000 simulated posterior estimates, each of which uses a randomly selected model set *M* and test set *N*. **Fig D in S1 File. Mode I: Identification of 100 bp vs. 200 bp DNA.** Bayesian posterior estimates *p*(100bp|**Θ**) and p(200bp|**Θ**) for test data sets of *N* points given a model based on *M* points. Data is bootstrapped from translocations of (a) 100 bp NoLimits DNA and (b) 200 bp NoLimits DNA (main text: Fig 2A and 2B) corresponding to the Gaussian mixture models shown in Figs Ba and Bb. Each point represents the average of 1000 simulated posterior estimates, each of which uses randomly selected (disjoint) model set *M* and test set *N*. **Fig E in S1 File. Mode I: Identification of 900 bp vs. 1000 bp DNA.** Bayesian posterior estimates *p*(900bp|**Θ**) and p(1000bp|**Θ**) for test data sets of *N* points given a model based on *M* points. Data is bootstrapped from translocations of (a) 900 bp NoLimits DNA and (b) 1000 bp NoLimits DNA (main text: Fig 2C and 2D) corresponding to the Gaussian mixture models shown in Figs Bc and Bd. Each point represents the average of 1000 simulated posterior estimates, each of which uses randomly selected (disjoint) model set *M* and test set *N*. **Fig F in S1 File. Mode II: Identification of 1000 bp vs 800+200 bp DNA.** (a) 1000 bp at 1 nM. (b) 1:1 ratio of 800 bp + 200 bp, total concentration 2 nM. (c) Gaussian mixture model fit, 1000 bp. (d) Gaussian mixture model fit, 800 bp + 200 bp. (e) Bayesian posterior estimate *p*(1000bp|**Θ**) for test data sets of *N* points given a model based on *M* points. (f) Bayesian posterior estimate *p*(800+200bp|**Θ**) for test data sets of *N* points given a model based on *M* points. Translocations for all samples were collected in a single nanopore (4.8 nm diameter, effective thickness ~7 nm) with a +300 mV bias relative to *trans* (open pore current: 13 nA). To facilitate visualization of population density, a random white noise offset below the acquisition rate of this data (-2 μs < Δ*t* < +2 μs, acquisition rate 250 kHz) has been added to each *t*_D_ in panels (a) and (b). Numerical simulations for panels (e) and (f) were bootstrapped from the data in panels (a) and (b), respectively. Each point represents the average of 1000 simulated posterior estimates, each of which uses randomly selected (disjoint) model set *M* and test set *N*. **Fig G in S1 File. Identification of *M*. *tuberculosis* H37Ra vs. H37Rv *mazG* Samples.** Bayesian posterior estimates *p*(H37Ra|**Θ**) and p(H37Rv|**Θ**) for test data sets of *N* points given a model based on *M* points. Data is bootstrapped from translocations of (a) Tuberculosis H37Ra and (b) H37Rv *mazG* restriction digested fragments as described in S1 File Sections 1 and 2. Each point represents the average of 1000 simulated posterior estimates, each of which uses randomly selected (disjoint) model set *M* and test set *N*. **Fig H in S1 File. Identification of *S*. *aureus* FPR3757 vs. HOU-MR *parC* Samples.** Bayesian posterior estimates *p*(FPR3757|**Θ**) and *p*(HOU-MR|**Θ**) for test data sets of *N* points given a model based on *M* points. Data is bootstrapped from translocations of (a) MRSA FPR3757 and (b) HOU-MR *parC* restriction digested fragments as described in S1 File Sections 1 and 2. Each point represents the average of 1000 simulated posterior estimates, each of which uses randomly selected (disjoint) model set *M* and test set *N*.
(PDF)
