# Supplementary material for: Genomic Pathogen Typing Using Solid-State Nanopores
Source: PLoS One. 2015 Nov 12;10(11):e0142944. doi: 10.1371/journal.pone.0142944 (PMC4643041; doi:10.1371/journal.pone.0142944)
Supplement: S1 File — Gene and primer sequences for tuberculosis and MRSA strains. Additional details for PCR and restriction digest (Fig A). Bayesian classification for Mode I: Additional data and numerical simulations (Figs B-E). Bayesian classification for Mode II: Additional data and numerical simulations (Fig F). M. tuberculosis and methicillin-resistant S. aureus SNV detection: Additional data and numerical simulations (Figs G and H). Fig A in S1 File. PCR and Restriction Digest Products. Native PAGE showing successful PCR amplification of the mazG gene (M. tuberculosis) and parC gene (S. aureus). Digestion reactions yield either cut or uncut amplicons depending upon the parent strain. Lane 1: loading dye. Lane 2: 100 bp NEB ladder. Lane 3: mazG gene amplified from H37Ra (942 bp). Lane 4: mazG gene amplified from H37Rv (942 bp). Lane 5: mazG from H37Ra after digestion with NaeI enzyme, not cut. Lane 6: mazG from H37Rv after digestion with NaeI, cut into two fragments of 321 and 621 bp. Lane 7: parC gene fragment amplified from HOU-MR strain (885 bp). Lane 8: parC gene fragment amplified from FPR3757 strain (885 bp). Lane 9: parC from HOU-MR after digestion reaction with BseRI, cut into two fragments of 245 and 640 bp. Lane 10: parC from FPR3757 after digestion reaction with BseRI, not cut. Digestion reactions were performed at 37°C for 1hr in NEB Cutsmart buffer. 10 units of enzyme were used for each digestion reaction. Fig B in S1 File. Gaussian Mixture Model Fits for DNA Translocation. Gaussian mixture model fits to translocations of single-length DNA samples through a 4.8 nm diameter nanopore (1M KCl, +300 mV bias). (a) 100 bp NoLimits DNA. (b) 200 bp NoLimits DNA. (c) 900 bp NoLimits DNA. (d) 1000 bp NoLimits DNA. Raw t D and ΔI data are shown in Fig 2 (main text). Fig C in S1 File. Bayesian Posterior Estimates for Nanopore Sample Identification. Bayesian posterior estimates p(100bp|Θ) and p(1000bp|Θ) for test data sets of N points given a model based on M points. Data is boots [file pone.0142944.s001.pdf]

Supporting Information and Figures:

Genomic Pathogen Typing Using Solid-State Nanopores

Allison H. Squires[1], Evrim Atas[1], and Amit Meller*[1,2]

[1]Department of Biomedical Engineering
Boston University
Boston, Massachusetts 02215
U.S.A.

[2]Department of Biomedical Engineering
The Technion – Israel Institute of Technology
Haifa, Israel 32000

*Corresponding author. E-mail ameller@bm.technion.ac.il

Table of Contents

1. Gene and primer sequences for *M. tuberculosis* and methicillin-resistant *S. aureus* strains
2. Additional details for PCR and restriction digest
3. Bayesian classification for Mode I: Additional data and numerical simulations
4. Bayesian classification for Mode II: Additional data and numerical simulations
5. *M. tuberculosis* and methicillin-resistant *S. aureus* SNV detection: Additional data and numerical simulations
6. References

1) Gene and primer sequences for *M. tuberculosis* and methicillin-resistant *S. aureus* strains

Target genes are PCR amplified from purified genomic DNA from *Mycobacterium tuberculosis* strains H37Ra (ATCC 25177) and H37Rv (ATCC 25618) and from methicillin-resistant *Streptococcus aureus* strains USA300-HOU-MR (also known as USA300-TCH1516) (ATCC BAA-1718) and USA300-FPR3757 (ATCC BAA-1556) freshly obtained from the American Type Culture Collection (ATCC).

Tuberculosis gene target: The *mazG* gene for both strains (978 bp long) of *M. tuberculosis* was targeted for SNV detection. The critical SNV is a point mutation from C to A at bp #624 in the *mazG* gene of the H37Ra strain, a non-synonymous mutation which is believed to have played a key role in the emergence of the avirulent strain by creating a competitive advantage for survival of aging-mediated cell lysis. With our designed primers, we obtained an amplicon of 942 bp. This amplicon contains only a single recognition site for the restriction enzyme NaeI (GCCGGC), which includes the site of the SNV [1]. NaeI can be used to digest the H37Rv amplicon into two fragments (621 bp and 321bp), and will not cut the H37Ra amplicon (942 bp). The amplicon, restriction enzyme recognition sequence, cut site, SNV, and primer sequences are shown below in the context of the *mazG* sequence from *M. tuberculosis* H37Rv (GenBank accession number: NC_000962.3, location Rv1021, bp #1142671-1143648) [2].

Key:
Amplicon = CAPS
Forward primer
Reverse primer
NaeI recognition site 5'-GCCGGC-3'
Cut site: \\
SNV (H37Rv: **C**, H37Ra: **A**)

***mazG* from *M. tuberculosis* H37Rv:**
5'-atgattgtcgtcctggtcgaccccggcgtcc**GACACTGGTGCCTGTTGAAG**CGATCGAGTTCCTGCGCGGCGAGGTGCA
ATACACCGAGGAAATGCCGGTCGCGGTGCCCTGGTCGCTACCAGCGGCTCGTTCGGCGCACGCCGGAAACGACG
CGCCGGTGTTGCTGTCGTCTGACCCCAACCATCCTGCTGTCATTACTCGACTGGCCGCCGGTGCCCGGCTGATCTC
GGCACCGGATTCTCAGCGTGGCGAACGACTCGTCGACGCCGTCGCGATGATGGACAAGCTGCGCACCGCCGGAC
CGTGGGAAAGTGAGCAGACTCACGACTCGCTGCGCAGATACCTGCTGGAGGAGACCTACGAGCTGTTGGACGCG
GTCCGCAGCGGCAGTGTTGACCAGCTGCGCGAAGAGCTTGGTGATCTCTTGCTGCAGGTCCTCTTTCACGCCCGGA
TCGCTGAGGATGCGTCGCAATCGCCGTTCACCATCGACGACGTCGCCGACACACTGATGCGAAAGCTCGGCAATC
GGGCGCCAGGAGTACTTGCGGGCGAATCGATTTCGCTCGAAGATCAACTGGCGCAATGGGAGGCAGCCAAGGCC
TCGGAAAAGGCGCGAAAGTCGGTAGCCGACGATGTCCATACGGGCCAGCC \\ GG**C**ATTAGCGCTGGCGCAGAA
GGTTATTCAGCGTGCCCAAAAGGCTGGGCTGCCCGCTCACCTGATCCCCGATGAGATCACTTCTGTTTCGGTTTCA
GCTGACGTAGATGCGGAAAACACGCTGCGCACTGCCGTTTTGGACTTTATTGACAGGCTGCGCTGTGCCGAGCGG
GCAATTGCCGTCGCACGCCGGGGCAGCAACGTTGCCGAGCAGCTCGATGTGACGCCGCTGGGTGTGATCACCGA
GCAGGAGTGGCTCGCGCATTGGCCAACTGCTGTCAACGATTCCCGCGGCGGGTC**CAAGAAACGTAAAGGCATGC**
Gataa-3'

MRSA gene target: The *parC* gene for both strains (2403 bp long) of methicillin-resistant *S. aureus* was targeted for SNV detection. The critical SNV is a point mutation from C to A at bp #239 in the *parC* gene of the FPR3757 strain, a non-synonymous mutation which is believed to confer fluoroquinolone

resistance [3]. With our designed primers, we obtained an amplicon of 885 bp. This amplicon contains only a single recognition site for the restriction enzyme BseRI (CTCCTC), which includes the site of the SNV [4]. Therefore, BseRI can be used to digest HOU-MR into two fragments (640 bp and 245 bp), and will not cut FPR3757 (885 bp). The amplicon, restriction enzyme recognition sequence, cut site, SNV, and primer sequences are shown below in the context of the *parC* gene from *S. aureus* HOU-MR [4].

Key:
parC gene start = ^
Amplicon = CAPS
Forward primer
Reverse Primer
BseRI recognition site 5'-CTCCTC-3'
Cut site: \\
SNV (USA300-HOU-MR : **C**, FPR3757: **A**)

***parC* from *S. aureus* USA300-HOU-MR:**
5'-ttt**GATGAGGAGGAAATCTA^GTG**AGTGAAATAATTCAAGATTTATCACTTGAAGATGTTTTAGGTGATCGCT
TTGGAAGATATAGTAAATATATTATTCAAGAGCGTGCATTGCCAGATGTTCGTGATGGTTTAAAACCAGTACAACG
TCGTATTTTATATGCAATGTATTCAAGTGGTAATACACACGATAAAAATTTCCGTAAAAGTGCGAAAACAGTCGGT
GATGTTATTGGTCAATATCATCCA \\ CATGGAGACT**C**CTCAGTGTACGAAGCAATGGTCCGTTTAAGTCAAGACTG
GAAGTTACGACATGTCTTAATAGAAATGCATGGTAATAATGGTAGTATCGATAATGATCCGCCAGCGGCAATGCG
TTACACTGAAGCTAAGTTAAGCTTACTAGCTGAAGAGTTATTACGTGATATTAATAAAGAGACAGTTTCTTTCATTC
CAAACTATGATGATACGACACTCGAACCAATGGTATTGCCATCAAGATTTCCTAACTTACTAGTGAATGGTTCTACA
GGTATATCTGCAGGTTACGCGACAGATATACCACCACATAATTTAGCTGAAGTGATTCAAGCAACACTTAAATATA
TTGATAATCCGGATATTACAGTCAATCAATTAATGAAATATATTAAAGGTCCTGATTTTCCAACTGGTGGTATTATT
CAAGGTATTGATGGTATTAAAAAAGCTTATGAATCAGGTAAAGGTAGAATTATAGTTCGTTCTAAAGTTGAAGAA
GAAACTTTACGCAATGGACGTAAACAGTTAATTATTACTGAAATTCCATATGAAGTGAACAAAAGTAGCTTAGTAA
AACGTATCGATGAATTACGTGCTGACAAAAAAGT**CGATGGTATCGTTGAAGTACGTG**atgaaactgatagaactggtttac
gaatagcaattgaattgaaaaaagatgtgaacagtgaatcaatcaaaaattatctttataaaaactctgatttacagatttcatataatttcaacatgg
tcgctattagtgatggtcgtccaaaattgatgggtattcgtcaaattatagatagttatttgaatcaccaaattgaggttgttgcaaatagaacgaagttt
gaattagataatgcagaaaaacgtatgcatatcgttgaaggtttgattaaagcgttgtcaattttagataaagtaatcgaattgattcgtagctctaaa
aacaagcgtgacgctaaagaaaaccttatcgaagtatacgagttcacagaagaacaggctgaagcaattgtaatgttacagttatatcgtttaacaa
atactgacatagttgcgcttgaaggtgaacataaagaacttgaagcattaatcaaacaattacgtcatattcttgataaccatgatgcattattgaatgt
cataaaagaagaattgaatgaaattaaaaagaaattcaaatctgaacgactgtctttaattgaagcagaaattgaagaaattaaaattgacaaaga
agttatggtgcctagtgaagaagttattttaagtatgacacgtcatggatatattaaacgtacttctattcgtagctttaatgctagcggtgttgaagata
ttggtttaaaagatggtgacagtttacttaaacatcaagaagtaaatacgcaagataccgtactagtatttacaaataaaggtcgttatctatttatacc
ggttcataaattagcagatattcgttggaaagaattgggacaacatgtatcacaaatagttcctatcgaagaagatgaagtggttattaatgtctttaat
gaaaaggactttaatacagatgcattttatgtttttgcgactcaaaatggcatgattaagaaaagtacagtgcctctatttaaaacaacgcgttttaata
aacctttaattgctactaaagttaaagaaaatgatgatttgattagtgttatgcgctttgaaaaagatcaattaattaccgtcattactaataaaggtat
gtcattaacgtataatacaagtgaactatcagataccggattaagggcagctggtgttaaatcaataaatcttaaagctgaagatttcgttgttatgac
agaaggtgtttctgaaaatgatactatattgatggccacacaacgcggctcgttaaaacgtattagttttaaaatcttacaagttgctaaaagagcaca
acgtggaataactttattaaaagaattaaagaaaaatccacatcgtattgtagctgcacatgtagtgacaggtgaacatagtcaatatacattatattc
aaaatcaaatgaagaacatggtttaattaatgatattcataaatctgaacaatatacaaatggctcattcattgtagatacagatgattttggtgaagt
aatagacatgtatattagctaa-3'

2) Additional details for PCR and restriction digest

Phusion DNA Polymerase was used to amplify targeted genes from the genomic DNA of both pathogens. We optimized the PCR conditions based on primer sequences. After successful PCR reactions, we tested the product sequences using specific digestion enzymes whose recognition sites overlapped with the SNV sites. For the *mazG* gene (TB), the NaeI restriction enzyme (NEB # R0190S) correctly cuts the amplicon into two fragments for the H37Rv strain only. H37Ra will remain uncut as it does not have the specific sequence for the digestion reaction. For *parC* gene (MRSA), BseRI (NEB # R0581S) correctly cuts the USA300-HOU-MR amplicon into two fragments, while the FPR3757 amplicon is uncut.

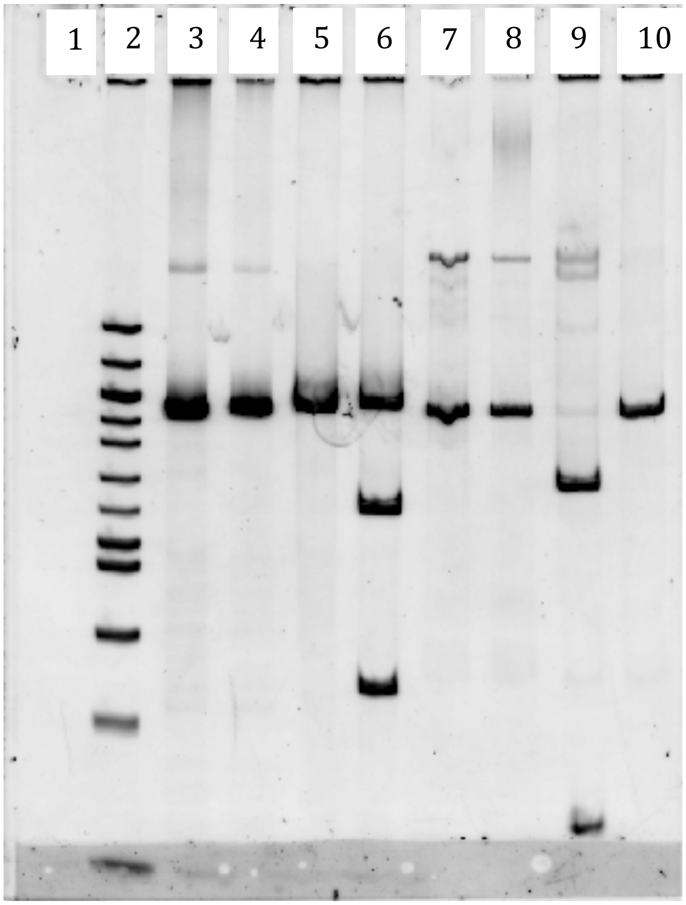

Fig A. **PCR and Restriction Digest Products.** Native PAGE showing successful PCR amplification of the *mazG* gene (*M. tuberculosis*) and *parC* gene (*S. aureus*). Digestion reactions yield either cut or uncut amplicons depending upon the parent strain. Lane 1: loading dye. Lane 2: 100 bp NEB ladder. Lane 3: *mazG* gene amplified from H37Ra (942 bp). Lane 4: *mazG* gene amplified from H37Rv (942 bp). Lane 5: *mazG* from H37Ra after digestion with NaeI enzyme, not cut. Lane 6: *mazG* from H37Rv after digestion with NaeI, cut into two fragments of 321 and 621 bp. Lane 7: *parC* gene fragment amplified from HOU-MR strain (885 bp). Lane 8: *parC* gene fragment amplified from FPR3757 strain (885 bp). Lane 9: *parC* from HOU-MR after digestion reaction with BseRI, cut into two fragments of 245 and 640 bp. Lane 10: *parC* from FPR3757 after digestion reaction with BseRI, not cut. Digestion reactions were performed at 37°C for 1hr in NEB Cutsmart buffer. 10 units of enzyme were used for each digestion reaction.

3) Bayesian classification for Mode I: Additional data and numerical simulations

Mode I detection requires direct length discrimination of fragments to determine the absence or presence of a large indel [5]. Figs B-E demonstrate the Bayesian classification scheme for different bootstrapping conditions on pairs of fragments as described in the main text. Fig B shows Gaussian mixture model fits for the data shown in Fig 2 (main text) for single-fragment samples. Figs C, D, and E quantify the posterior probabilities for correct classification of different length comparisons (C: 100 vs. 1000 bp, D: 100 vs. 200 bp, and E: 900 vs. 1000 bp).

As discussed in the main text, it is clear from these figures that it is relatively easy (M and N need be only a few translocations each) to distinguish 100 bp from 1000 bp with >95% confidence, and also easy to distinguish 100 bp from 200 bp. However, to distinguish the same Δl = 100 bp when l_0 = 900 bp (Fig E) is relatively difficult, and requires more points in both the model set and the test set for 95% confidence (M and N must contain at least several hundred points each). This illustrates the practical sample length limits on Mode I sensing: as the size of the indel Δl shrinks, or as the required base length l_0 grows, it becomes more difficult to confidently detect an indel based on fragment sizing alone. In this limit, Mode II sensing provides greater classification confidence for far fewer translocations.

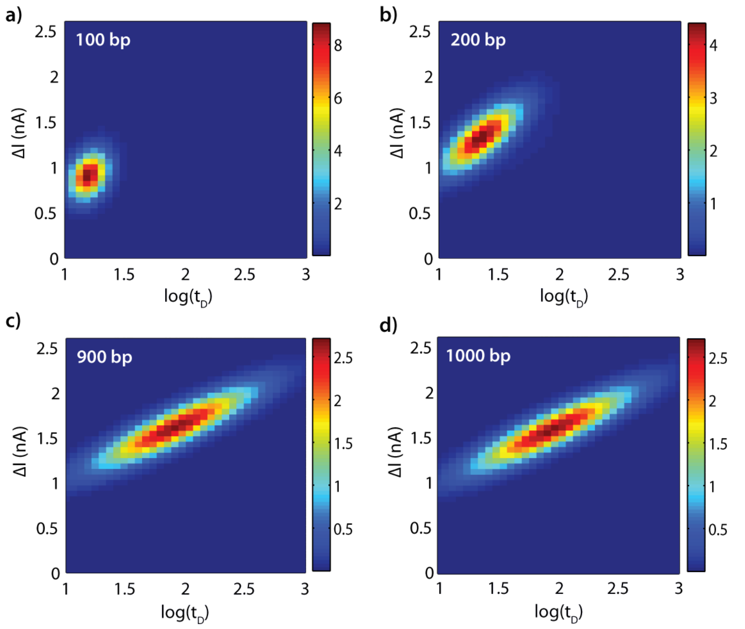

Fig B. Gaussian Mixture Model Fits for DNA Translocation. Gaussian mixture model fits to translocations of single-length DNA samples through a 4.8 nm diameter nanopore (1M KCl, +300 mV bias). (a) 100 bp NoLimits DNA. (b) 200 bp NoLimits DNA. (c) 900 bp NoLimits DNA. (d) 1000 bp NoLimits DNA. Raw t_D and ΔI data are shown in Fig 2 (main text).

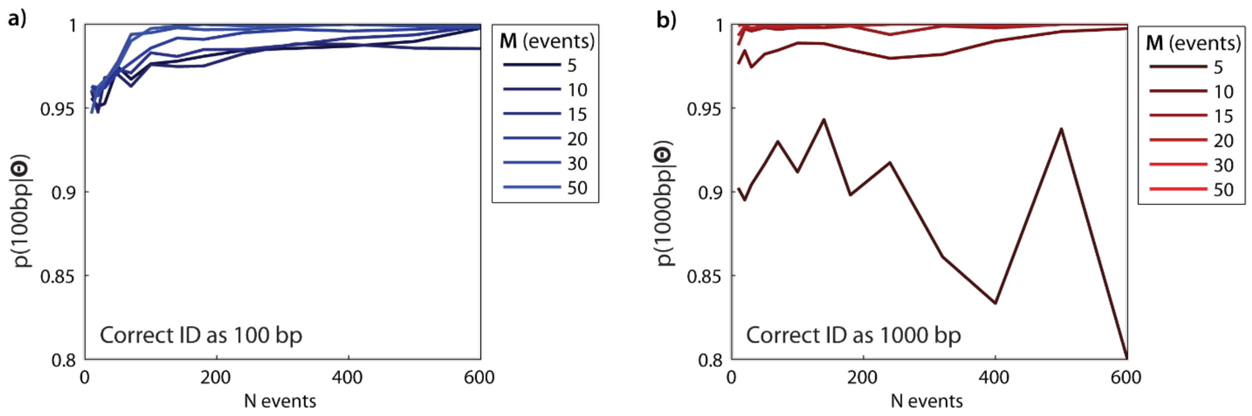

Fig C. Bayesian Posterior Estimates for Nanopore Sample Identification. Bayesian posterior estimates *p*(100bp|**Θ**) and p(1000bp|**Θ**) for test data sets of *N* points given a model based on *M* points. Data is bootstrapped from translocations of (a) 100 bp NoLimits DNA and (b) 1000 bp NoLimits DNA (main text: Figs 2a and 2d) corresponding to the Gaussian Mixture Models shown in Figs Ba and Bd. Each point represents the average of 1000 simulated posterior estimates, each of which uses a randomly selected model set *M* and test set *N*.

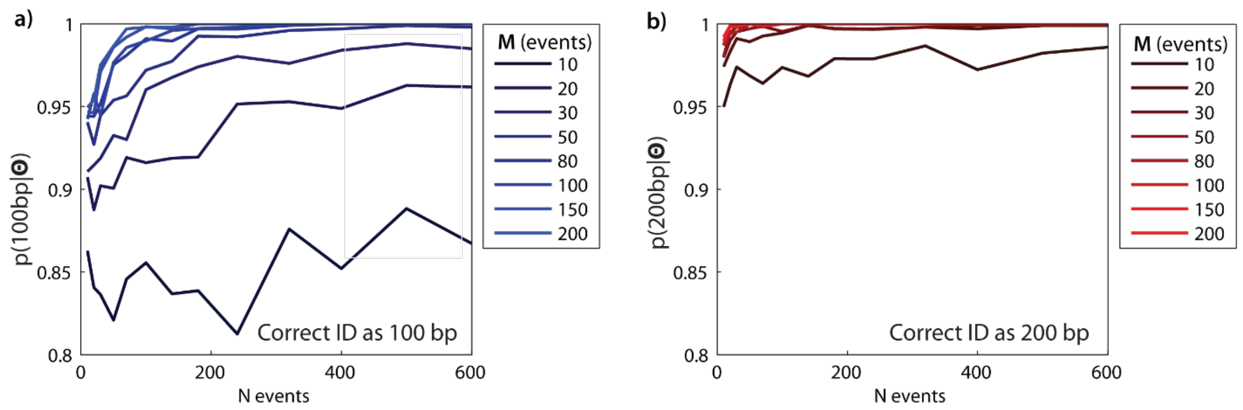

Fig D. Mode 1: Identification of 100 bp vs. 200 bp DNA. Bayesian posterior estimates *p*(100bp|**Θ**) and p(200bp|**Θ**) for test data sets of *N* points given a model based on *M* points. Data is bootstrapped from translocations of (a) 100 bp NoLimits DNA and (b) 200 bp NoLimits DNA (main text: Figs 2a and 2b) corresponding to the Gaussian mixture models shown in Figs Ba and Bb. Each point represents the average of 1000 simulated posterior estimates, each of which uses randomly selected (disjoint) model set *M* and test set *N*.

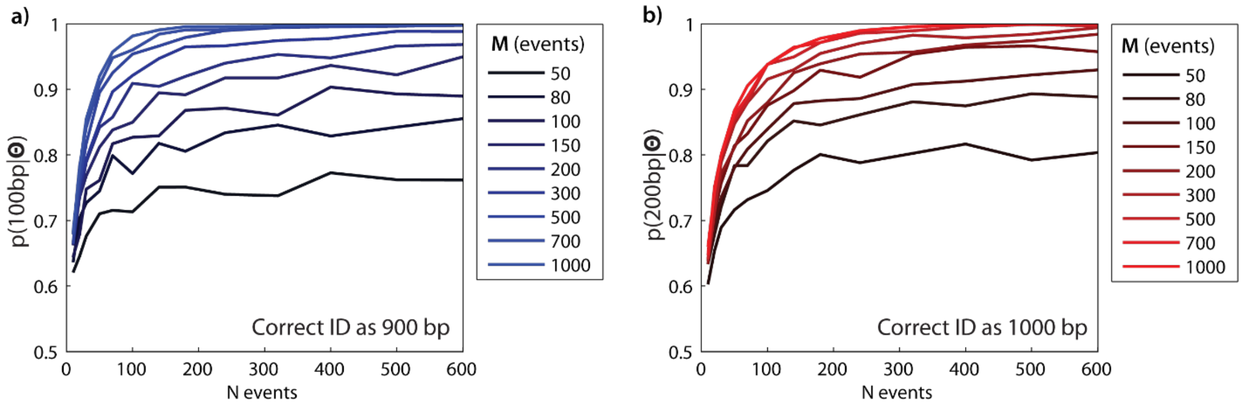

Fig E. Mode I: Identification of 900 bp vs. 1000 bp DNA. Bayesian posterior estimates *p*(900bp|**Θ**) and p(1000bp|**Θ**) for test data sets of *N* points given a model based on *M* points. Data is bootstrapped from translocations of (a) 900 bp NoLimits DNA and (b) 1000 bp NoLimits DNA (main text: Figs 2c and 2d) corresponding to the Gaussian mixture models shown in Figs Bc and Bd. Each point represents the average of 1000 simulated posterior estimates, each of which uses randomly selected (disjoint) model set *M* and test set *N*.

4) Bayesian classification for Mode II: Additional data and numerical simulations

Additional data showing discrimination of 1000 bp from a slightly different asymmetric cut site (800 + 200 bp) is shown in Fig F.

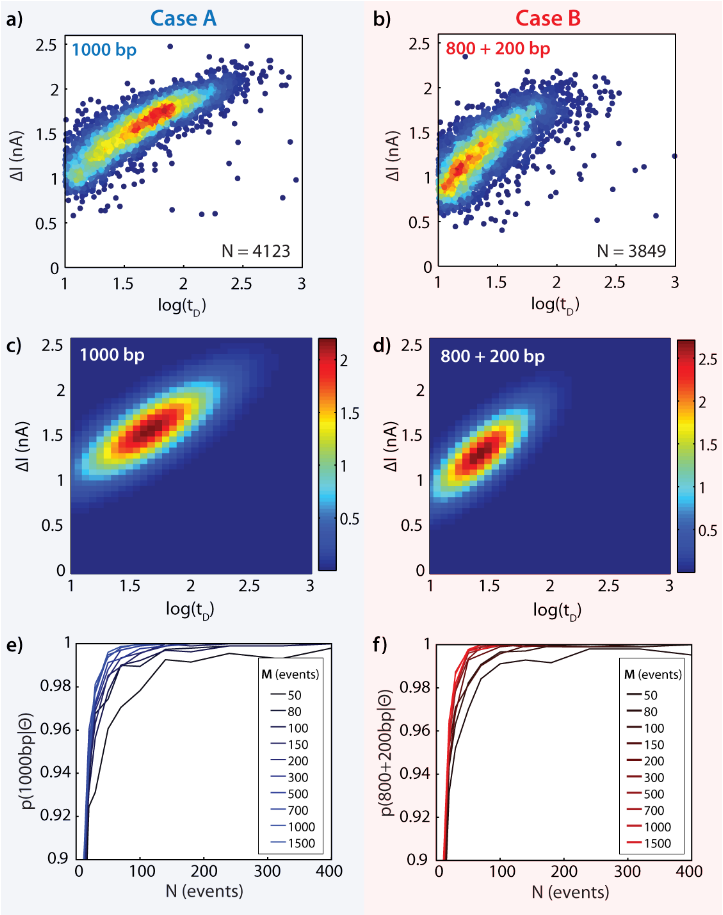

Fig F. Mode II: Identification of 1000 bp vs. 800+200 bp DNA. (a) 1000 bp at 1 nM. (b) 1:1 ratio of 800 bp + 200 bp, total concentration 2 nM. (c) Gaussian mixture model fit, 1000 bp. (d) Gaussian mixture model fit, 800 bp + 200 bp. (e) Bayesian posterior estimate $p(1000bp|\mathbf{\Theta})$ for test data sets of N points given a model based on M points. (f) Bayesian posterior estimate $p(800{+}200bp|\mathbf{\Theta})$ for test data sets of N points given a model based on M points. Translocations for all samples were collected in a single nanopore (4.8 nm diameter, effective thickness ~7 nm) with a +300 mV bias relative to *trans* (open pore current: 13 nA). To facilitate visualization of population density, a random white noise offset below the acquisition rate of this data (-2 μs < Δt < +2 μs, acquisition rate 250 kHz) has been added to each t_D in panels (a) and (b). Numerical simulations for panels (e) and (f) were bootstrapped from the data in panels (a) and (b), respectively. Each point represents the average of 1000 simulated posterior estimates, each of which uses randomly selected (disjoint) model set M and test set N.

5) *M. tuberculosis* and methicillin-resistant *S. aureus* SNV detection: Additional data and numerical simulations

Fig 4 (main text) shows posterior estimates for Mode II type sensing to differentiate two strains of Tuberculosis (H37Ra and H37Rv) and MRSA (FPR3757 and HOU-MR) based only on a single nucleotide variation. Figs G and H show the dependence of these posterior estimates on the number of points used to fit the Gaussian mixture model, *M*. In both cases, only a few tens of points in the model (*M*) are required to reach 95% classification confidence using only a few tens of test points (*N*). The data presented in Fig 4 (main text) is based on *M*=100, boxed here in red on each panel.

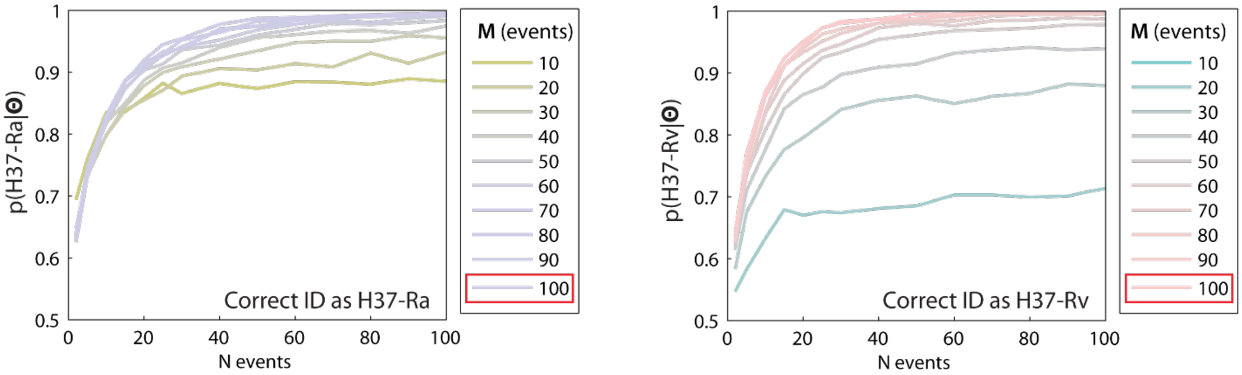

Fig G. Identification of *M. tuberculosis* H37Ra vs. H37Rv *mazG* Samples. Bayesian posterior estimates *p*(H37Ra|**Θ**) and p(H37Rv|**Θ**) for test data sets of *N* points given a model based on *M* points. Data is bootstrapped from translocations of (a) Tuberculosis H37Ra and (b) H37Rv *mazG* restriction digested fragments as described in S1 File Sections 1 and 2. Each point represents the average of 1000 simulated posterior estimates, each of which uses randomly selected (disjoint) model set *M* and test set *N*.

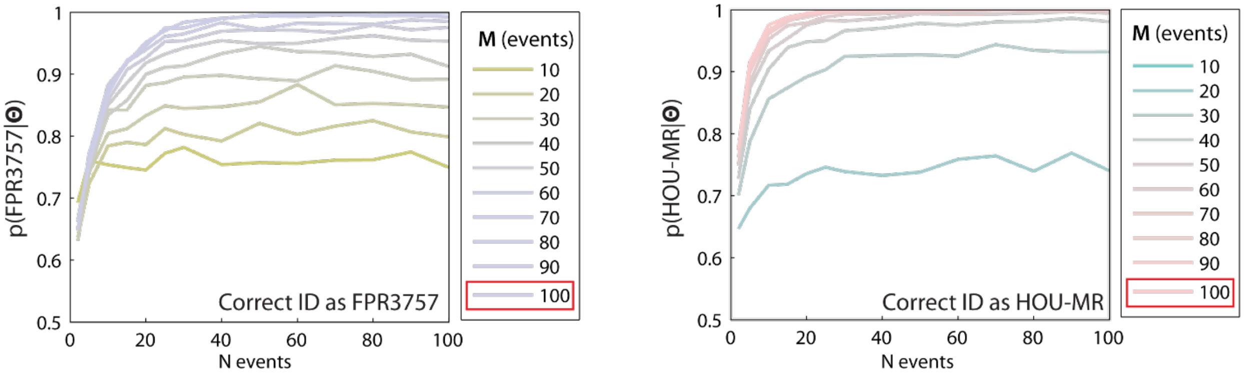

Fig H. Identification of *S. aureus* FPR3757 vs. HOU-MR *parC* Samples. Bayesian posterior estimates *p*(FPR3757|**Θ**) and *p*(HOU-MR|**Θ**) for test data sets of *N* points given a model based on *M* points. Data is bootstrapped from translocations of (a) MRSA FPR3757 and (b) HOU-MR *parC* restriction digested fragments as described in S1 File Sections 1 and 2. Each point represents the average of 1000 simulated posterior estimates, each of which uses randomly selected (disjoint) model set *M* and test set *N*.

6) References

1. Zheng H, Lu L, Wang B, Pu S, Zhang X, Zhu G, et al. Genetic basis of virulence attenuation revealed by comparative genomic analysis of *Mycobacterium tuberculosis* strain H37Ra versus H37Rv. PLoS One. 2008;3(6):e2375. Epub 2008/06/28. doi: 10.1371/journal.pone.0002375. PubMed PMID: 18584054; PubMed Central PMCID: PMC2440308.
2. Cole ST, Brosch R, Parkhill J, Garnier T, Churcher C, Harris D, et al. Deciphering the biology of *Mycobacterium tuberculosis* from the complete genome sequence. Nature. 1998;393(6685):537-44. Epub 1998/06/20. doi: 10.1038/31159. PubMed PMID: 9634230.
3. Ferrero L, Cameron B, Crouzet J. Analysis of gyrA and grlA mutations in stepwise-selected ciprofloxacin-resistant mutants of *Staphylococcus aureus*. Antimicrob Agents Ch. 1995;39(7):1554-8. Epub 1995/07/01. PubMed PMID: 7492103; PubMed Central PMCID: PMC162780.
4. Highlander SK, Hulten KG, Qin X, Jiang H, Yerrapragada S, Mason EO, Jr., et al. Subtle genetic changes enhance virulence of methicillin resistant and sensitive *Staphylococcus aureus*. BMC Microbiol. 2007;7:99. Epub 2007/11/08. doi: 10.1186/1471-2180-7-99. PubMed PMID: 17986343; PubMed Central PMCID: PMC2222628.
5. Duda RO, Hart PE, Stork DG. Pattern Classification (2nd Edition): Wiley-Interscience; 2000.
